# Taking advantage of hybrid bioinspired intelligent algorithm with decoupled extended Kalman filter for optimizing growing and pruning radial basis function network

**DOI:** 10.1098/rsos.180529

**Published:** 2018-09-19

**Authors:** Zhilei Chai, Wei Song, Qinxin Bao, Feng Ding, Fei Liu

**Affiliations:** 1 School of Internet of Things (IOT) Engineering, Jiangnan University, Wuxi, Jiangsu, China; 2 Jiangsu Provincial Engineering Laboratory of Pattern Recognition and Computational Intelligence, Jiangnan University, Wuxi, Jiangsu, China; 3 Engineering Research Center of Internet of Things Applied Technology, Ministry of Education, Wuxi, Jiangsu, China

**Keywords:** bioinspired intelligent algorithm, prediction analysis, competitive mechanism, GAP-RBF

## Abstract

The growing and pruning radial basis function (GAP-RBF) network is a promising sequential learning algorithm for prediction analysis, but the parameter selection of such a network is usually a non-convex problem and makes it difficult to handle. In this paper, a hybrid bioinspired intelligent algorithm is proposed to optimize GAP-RBF. Specifically, the excellent local convergence of particle swarm optimization (PSO) and the extensive search ability of genetic algorithm (GA) are both considered to optimize the weights and bias term of GAP-RBF. Meanwhile, a competitive mechanism is proposed to make the hybrid algorithm choose the appropriate individuals for effective search and further improve its optimization ability. Moreover, a decoupled extended Kalman filter (DEKF) method is introduced in this study to reduce the size of error covariance matrix and decrease the computational complexity for performing real-time predictions. In the experiments, three classic forecasting issues including abalone age, Boston house price and auto MPG are adopted for extensive test, and the experimental results show that our method performs better than PSO and GA these two single bioinspired optimization algorithms. What is more, our method via DEKF achieves the better results in comparison with the state-of-art sequential learning algorithms, such as GAP-RBF, minimal resource allocation network, resource allocation network using an extended Kalman filter and resource allocation network.

## 1. Introduction

Radial basis function (RBF) network is well known in recent years due to its ability to solve complex nonlinear problems with a single-layered neural network. The attractiveness of RBF topology comes as a result of the high accuracy and generalization property when it faces complex engineering problems [1]. In the past decade, the research of RBF has witnessed a number of successful applications, such as classification [2], regression [3], system identification [4] and pattern recognition [5].

In comparison with multilayer perceptron networks, the basic RBF network has a simple topological structure with only one hidden layer using Gaussian basis function and one output layer with the linear combination of basis functions and connecting weights. For the classical implementations, the number of hidden units is fixed *a priori* based on some properties of input data [6]. The weights connecting hidden and output units are usually estimated by linear least squares, such as least mean square (LMS) [7,8] and recursive least squares [9]. As we know, these classical approaches are not very suitable for sequential learning and usually result in too many hidden units [10]. To deal with this issue, resource allocation network (RAN) was proposed by Platt [11] through adding hidden neurons sequentially if a ‘novelty’ is satisfied to new input data. Otherwise, the network is adjusted by a standard LMS to fit the data. Kadirkamanathan & Nirajan [12] enhanced resource allocation network using an extended Kalman filter (RANEKF), instead of LMS, to update the network. However, the number of hidden neurons would expand abruptly with the increase of the observed data. What is more, some hidden units, although active initially, may subsequently end up owing to contributing little to the network output [6]. Yingwei *et al*. [13] improved RAN and proposed a minimal resource allocation network (MRAN) through introducing smooth growing and pruning strategies. In MRAN, the distribution of the input data would directly affect the selection for the appropriate size of sliding windows. It is interesting to note that all the above algorithms do not link the required accuracy directly to the learning process. Instead, they have a series of thresholds needed to select by exhaustive simulation trials. Huang *et al*. [14] presented a sequential learning algorithm through estimating the significance of each neuron as the criterion for growing and pruning radial basis function (GAP-RBF) neurons. The GAP-RBF algorithm would ensure that a neuron will not be added if it is not significant to the overall performance of the network, even though it may make contribution to the single latest input data. Such an algorithm effectively overcomes the issue by directly linking the desired accuracy to the learning algorithm and provides higher generalization performance with reduced computational complexity. In comparison with previous RANs, GAP-RBF has very few thresholds to define and requires less memory. However, when using GAP-RBF to deal with real-world problems, it cannot achieve a better accuracy than MRAN and RANEKF [14]. That is because the significance condition is difficult to precisely estimate, especially for non-uniform distribution of input data, which causes the issue of inaccurate selection for the parameters of GAP-RBF.

It has been known that the parameter selection of network to deal with real-world problems is usually a complex and non-convex problem, where global optima would be difficult to handle by gradient-based optimization algorithms. Bioinspired intelligent algorithms, e.g. particle swarm optimization (PSO) [15] and genetic algorithm (GA) [16], have received special attention in recent decades due to their capacity to solve problems that cannot be effectively solved by traditional optimization algorithms. For instance, RBF has been optimized by PSO [17] and GA [18,19]. However, although PSO and GA are versatile and popular, the search ability of single bioinspired optimization algorithm is still restricted by its simple and initial conception. For instance, PSO may get trapped at local optima, and GA may be relatively inefficient for local search. In this paper, a hybrid bioinspired intelligent algorithm is proposed. Specifically, the excellent local convergence of PSO and extensive search ability of GA are both considered to optimize the weights and bias term between hidden layer and output layer, which are difficult to precisely optimize in basic GAP-RBF. Meanwhile, a competitive mechanism is introduced based on the respective advantage of PSO and GA to improve the search ability of the hybrid algorithm. That is, the population is partitioned in terms of the fitness of individual, and the hybrid method can choose the appropriate individuals for effective search. What is more, as we know, the large number of inputs may cause the rapid growth of neurons and makes the covariance matrix become huge. Such an undesirable process would finally result in an issue of computational overload [20]. Decoupled extended Kalman filter (DEKF) is introduced in this study by ignoring the cross-correlation terms between the nearest neuron and all other neurons in the error covariance matrix. Such a method reduces the size of covariance matrix and improves the performance of our network.

The rest of this paper is organized as follows. Section 2 gives a brief description of the related work including original GAP-RBF, PSO and GA. In §3, a hybrid bioinspired intelligent algorithm is proposed combining with a competitive mechanism to enhance its search ability to optimize GAP-RBF. Meanwhile, DEKF is introduced in this section to improve the computational efficiency of our network. In §4, three real-world benchmark problems are extensively tested to demonstrate the performance of our method. Section 5 concludes this study.

## 2. Related work

### 2.1. Growing and pruning radial basis function network

GAP-RBF is a promising feed-forward neural network proposed by Huang *et al*. [14]. Such a network introduces the concept of significance with respect to each neuron and uses it for growing and pruning hidden neurons. The neuron significance is defined as the contribution made by that neuron to the network output averaged over all the knowledge of input data received so far. The output of an RBF network with respect to an input vector *x* ∈ *R^l^* is given by:

2.1 $$f(x)=\alpha_{0}+\sum_{k=1}^{K}\alpha_{k}\phi_{k}(x),$$

where *K* is the number of neurons, *α*_0_ and *α_k_* are bias term and connecting weight vector to the output neurons, respectively. *ϕ_k_*(*x*) is the Gaussian response of the *k*th hidden neuron:

2.2 $$\phi_{k}(x)=\exp\left(-\frac{{∥x-\mu_{k}∥}^{2}}{\sigma_{k}^{2}}\right),$$

where *μ_k_* ∈ *R^l^* and *σ_k_* are the centre and width of the *k*th hidden neuron, respectively. The learning steps of GAP-RBF contain the allocation of new hidden neurons, adaptation of network parameters as well as pruning neurons of few contributions. The network begins with no hidden neuron. When a new observation (*x_n_*, *y_n_*) is received during training, a new hidden neuron will be allocated if the growing condition is satisfied. The growing condition is given as:

2.3 $$\{\begin{array}{c} ∥x_{n}-\mu_{nr}∥>\varepsilon_{n},\\ ∥e_{n}∥>e_{\min},\\ E_{\mathrm{sig}}(K+1)\;\;\;>e_{\min}. \end{array}$$

where *e_n_* = *y_n_* − *f*(*x_n_*) denotes the error between the real output and expected output of the network. *μ_nr_* is the nearest centre to *x_n_*. The value of *e*_min_ represents the desired approximation accuracy of the network output, and *ɛ_n_* distance is the scale of resolution in the input space. The algorithm begins with *ɛ_n_* = *ɛ*_max_, and *ɛ*_max_ is chosen as the largest scale of interest in the input space, typically the entire input space of non-zero probability. *ɛ_n_* distance is decayed exponentially as *ɛ_n_* = {*ɛ*_max_*γ^n^*, *ɛ*_min_}, where 0 < *γ* < 1 is a decay constant [13], and the value of *ɛ_n_* is decayed until it reaches *ɛ*_min_ [6]. Specifically, GAP-RBF derived the significance using a piecewise linear approximation to the Gaussian functions for the sake of reducing the computational efforts. Subsequently, the significance of the *k*th hidden neuron *E*_sig_(*k*) is estimated as

2.4 $$E_{\mathrm{sig}}(k)=\left|\frac{{\left(1.8\sigma_{k}\right)}^{l}\alpha_{k}}{S(X)}\right|,$$

where *S*(*X*) is the size of the input space, and *l* is the dimension of the input space. Thus, when all of these three criteria in formula (2.3) are satisfied, a new neuron *K* + 1 is added to the network as:

2.5 $$\{\begin{array}{c} \alpha_{K+1}=e_{n},\;\\ \mu_{K+1}=x_{n},\;\\ \sigma_{K+1}=\kappa ∥x_{n}-\mu_{nr}∥\; \end{array}$$

where *κ* is an overlap factor determining the amount of overlap with respect to the response of hidden units in the input space. If an observation (*x_n_*, *y_n_*) does not meet all of the three criteria in formula (2.3), only the network parameters of the nearest neuron to the current input are adapted using the extended Kalman filter (EKF) algorithm to fit that observation. Finally, to keep a compact network, the nearest neuron is checked for pruning according to its significance *E*_sig_(*nr*) which is given by:

2.6 $$E_{\mathrm{sig}}(nr)=\frac{|\alpha_{nr}|{\left(1.8\sigma_{nr}\right)}^{l}}{S(X)}<e_{\min}.$$

If the average contribution made by neuron *nr* in the whole range *X* is less than the expected accuracy *e*_min_, that is to say, neuron *nr* is insignificant, and this neuron will be removed [21]. In comparison with previous RANs, GAP-RBF has very few thresholds to define, but when using GAP-RBF to deal with real-world problems, it cannot achieve a better accuracy, because the significance condition is difficult to precisely estimate, especial for non-uniform distribution of input data.

### 2.2. Bioinspired intelligent algorithms

As we know, the optimization of network to tackle with real-world problems is usually complex and non-convex issue. In such a situation, it is difficult to obtain the global optima or near global optima using traditional gradient-based optimization algorithms. Inspired by the rules of biological intelligence, swarm sociality or natural phenomena, bioinspired intelligent algorithms have received extensive attention since they can effectively resolve the above issue.

As one of typical bioinspired intelligent algorithms, PSO was proposed by Kennedy & Eberhart [15,22] based on the concepts of social models and swarm theories. The swarm consists of individual particles, and it assumes that each particle in the swarm flies over the search space looking for promising regions of the landscape [23]. In each iteration, each particle moves towards the personal best solution and the global best solution concurrently to explore the optimum solution in the search space [24]. PSO is established based on few or even no assumption on the search space. Such a feature enables PSO to search the optimum solution in a wide search space. Though using the best information of individual and swarm, PSO shares relatively comprehensive search ability to an optimization problem. However, it performs poorly on problems that have many potential optima and may get trapped at local optima [25].

GA was introduced by Holland [16] as a powerful bioinspired intelligent algorithm for global search and optimization. It is a random searching algorithm based on computational models simulating the evolutionary mechanism of nature, and can solve nonlinear problems by searching all spaces [26]. GA has become more popular because of its relative simplicity and robustness [27]. In the population, each chromosome evolves in parallel, repeatedly modifying individual solutions. Though GA shows strong global search ability, the blindness of random crossover and mutation operators makes it difficult to perform detailed search for local optima. Thus, through combing other powerful search algorithms for evolving the global optimum solution, it has become the recent research hot spot for GA. Note that PSO uses the individual and social best information and shares the excellent local convergence. In this study, the extensive search ability of GA and the excellent local convergence of PSO are both considered to optimize the weights and bias term between hidden layer and output layer, which are difficult to precisely optimize in basic GAP-RBF.

## 3. A competitive mechanism-based hybrid bioinspired intelligent algorithm with decoupled extended Kalman filter for optimizing growing and pruning radial basis function network

In GAP-RBF network, weights and bias term are two kinds of important parameters which directly affect the performance of the network. However, in basic GAP-RBF the significance condition is difficult to precisely estimate and causes the issue of inaccurate selection for these two kinds of parameters. In this paper, a hybrid bioinspired intelligent algorithm considering the advantages of GA and PSO is proposed in conjunction with a competitive mechanism to optimize the weights and bias term of GAP-RBF. Specifically, the initial structure of GAP-RBF network is firstly obtained after the input of all observations, and then our method is used to further optimize the weights and bias term on basis of the historic input observations.

### 3.1. A hybrid bioinspired intelligent algorithm based on competitive mechanism

As we know, the inappropriate selection of weights and bias term would degrade the validity and accuracy of GAP-RBF. In this study, hybrid bioinspired intelligent algorithm based on competitive mechanism (HBIACM) is proposed to improve the performance of the network. In view of the similar individual structures, i.e. chromosomes corresponding to GA and particles corresponding to PSO, an initial population including these two kinds of individuals is randomly generated. In this population, weights and bias term between hidden layer and output layer of GAP-RBF are encoded to make up each individual. In order to guide the evolution direction of the population, fitness function is defined to evaluate the quality of each individual. That is to say, the fitness function will guide the population to seek the optimal weights and bias term, and causes the decrease of output error. In this study, mean square error (MSE) function [28] is used as the fitness function which is defined as

**Figure 1.**
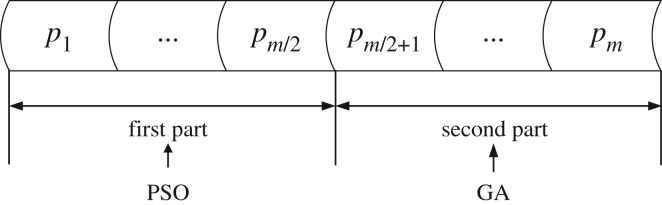
Population sorting for HBIACM.

3.1 $$\text{fit}=\text{MSE}=\frac{\sum_{i=1}^{n}{∥e_{i}∥}^{2}}{n},$$

where *e_i_* is the error between the experimental output and the expected output. Note that lower MSE would cause smaller fitness, which represents the relatively good individual. As shown in figure 1, the population of *m* individuals is ordered as fit(*p*_1_) ≤ fit(*p*_2_) ≤ … ≤ fit(*p_m_*_/2_) ≤ fit(*p_m_*_/2+1_) ≤ … ≤ fit(*p_m_*), and then we divide the whole population into two parts with the same size where PSO and GA act on the first and second regions, respectively.

The individuals at the first part of the population after sorting represent the good global optimum candidates. For this reason, as the excellent local convergence optimization algorithm, PSO is applied to this part. On the contrary, the individuals at the second part of population have little possibility of getting good fitness evaluation, which makes them relatively bad global optimum candidates. Thus, the crossover and mutation operators of GA are used to perform extensively exploration. Moreover, the utilization of crossover and mutation to the second part can effectively prevent destroying good individuals in the first part. Thus, the steps of HBIACM are described as follows:

*Step 1*: HBIACM initializes the population elements, and counter *c*_1_ recording the number of algorithm iterations is set to 0.
*Step 2*: The individuals are sorted with ascending order by their fitness values, and the first individual is selected as the current global optimum solution.
*Step 3*: PSO is performed on each element of the first part of population, and GA is applied to each element of the second part of population.
*Step 4*: Increase the iteration counter *c*_1_ if it does not exceed the upper bound, then go to Step 2. Otherwise, the algorithm is terminated. The achieved lowest element is the desired optimum solution to the current network structure.

### 3.2. Updating parameters with decoupled extended Kalman filter

Through considering the advantages of GA and PSO, HBIACM is proposed in conjunction with the competitive mechanism to optimize the weights and bias term of GAP-RBF. Such a method also brings extra computational cost and causes increased calculation time. Moreover, if the sample characteristics are complex, GAP-RBF would require a large number of hidden neurons resulting in very long training time and increased memory space [20]. In order to decrease the training time, we present a DEKF method, instead of EKF in GAP-RBF, to update the parameters of the network. At the time step *t* + 1 of the original EKF, the error covariance matrix *P* is adapted by:

3.2 $$K(t)=P(t)B(t){\left[R(t)+B^{T}(t)P(t)B(t)\right]}^{-1},$$

3.3 $$P(t+1)=[I-K(t)B^{T}(t)]P(t)+Q(t)I,$$

where *K*(*t*) is the Kalman gains vector, *R*(*t*) is the variance of the measurement noise, *B*(*t*) is the partial derivatives for the output signal and *Q*(*t*) is the artificial process noise added to avoid convergence to local optimum. Thus, in the original EKF method when the parameters of the nearest neuron need to be adjusted, all the cross-correlation terms between this nearest neuron and all other neurons in error covariance matrix would be updated together as shown below:

3.4 $$P(t)=\left[\begin{array}{cccccc} P_{11}(t)\; & P_{12}(t)\; & \cdots \; & P_{1nr}(t)\; & \cdots \; & P_{1N}(t)\;\\ P_{21}(t)\; & P_{22}(t)\; & \cdots \; & P_{2nr}(t)\; & \cdots \; & P_{2N}(t)\;\\ \vdots \; & \vdots \; & \ddots \; & \vdots \; & \ddots \; & \vdots \;\\ P_{nr1}(t)\; & P_{nr2}(t)\; & \cdots \; & P_{nrnr}(t)\; & \cdots \; & P_{nrN}(t)\;\\ \vdots \; & \vdots \; & \ddots \; & \vdots \; & \ddots \; & \vdots \;\\ P_{N1}(t)\; & P_{N2}(t)\; & \cdots \; & P_{Nnr}(t)\; & \cdots \; & P_{NN}(t)\; \end{array}\right],$$

where *N* is the current number of hidden neurons. Except the relevant elements to the nearest neuron, the rest of the elements in error covariance matrix *P* are maintained the same as their previous values. It has been known that when the structure of RBF network is small, the original EKF method is efficient. However, when the number of neurons becomes large or the input dimension is high, as a result the cross-correlation terms in the error covariance matrix *P* would be very huge and result in very high time complexity. In order to decrease training time, we use DEKF method which ignores the interdependencies of mutually exclusive groups of neurons. That is because the first and the second derivatives of Gaussian function *ϕ*(*x*) in equation (2.2) would approach zero much faster as input vector *x* moves away from the nearest centre *μ* in equation (2.2). Thus, all the cross-correlation terms between this nearest neuron and all other neurons would approach zero quickly. That is, the parameters of the nearest neuron are decoupled from other neurons, and the cross-correlation terms would have little effect on updating parameters. As a consequence, an improved method of DEKF is used in this study to reduce the computational burden and increase the learning speed. Specifically, when the nearest neuron is updated, the cross-correlation elements to all the other neurons in the error covariance matrix *P* are regarded as 0. Thus, the improved *P*(*t*) matrix is given as:

3.5 $$P(t)=\left[\begin{array}{cccccc} P_{11}(t)\; & 0\; & \cdots \; & 0\; & \cdots \; & 0\;\\ 0\; & P_{22}(t)\; & \cdots \; & 0\; & \cdots \; & 0\;\\ \vdots \; & \vdots \; & \ddots \; & \vdots \; & \ddots \; & \vdots \;\\ 0\; & 0\; & \cdots \; & P_{nrnr}(t)\; & \cdots \; & 0\;\\ \vdots \; & \vdots \; & \ddots \; & \vdots \; & \ddots \; & \vdots \;\\ 0\; & 0\; & \cdots \; & 0\; & \cdots \; & P_{NN}(t)\; \end{array}\right].$$

We can see in the improved *P*(*t*) matrix that only *P_nrnr_*(*t*) element needs to be updated. Thus, the computational time and memory requirement are greatly reduced. Subsequently, the pseudo code of the training steps of our algorithm is given as:


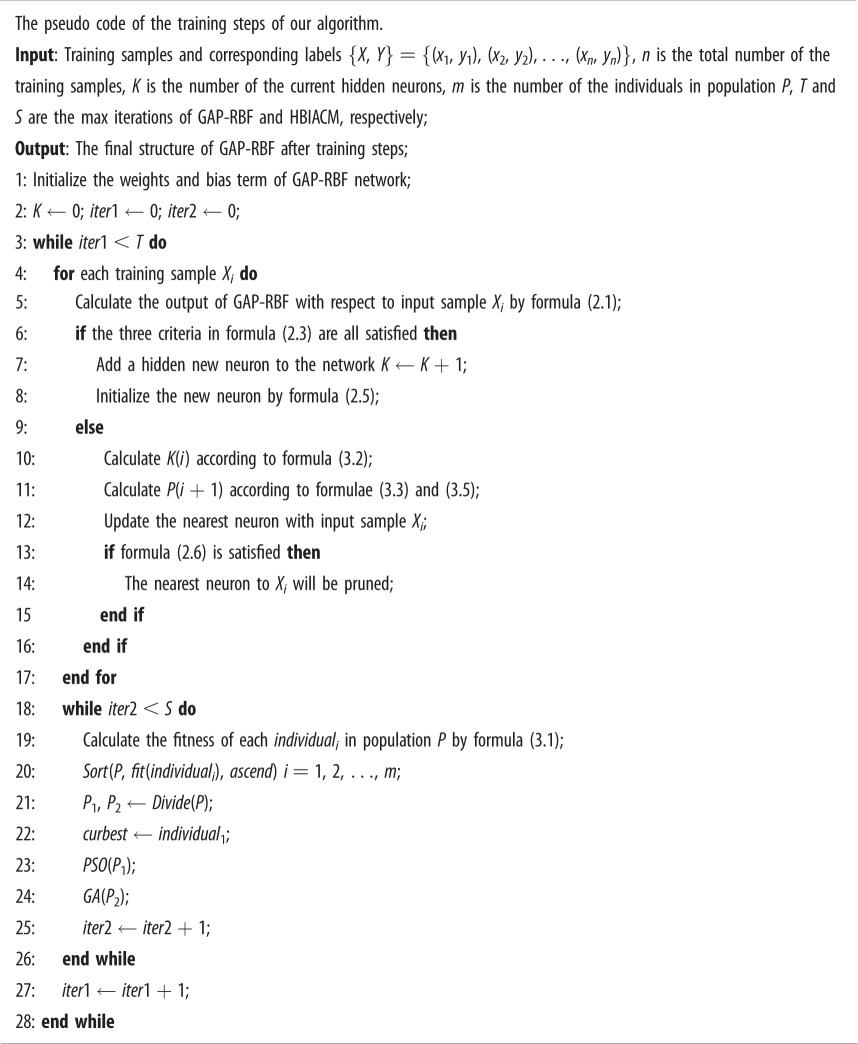


Finally, the pseudo code of the testing steps of our algorithm is given as:


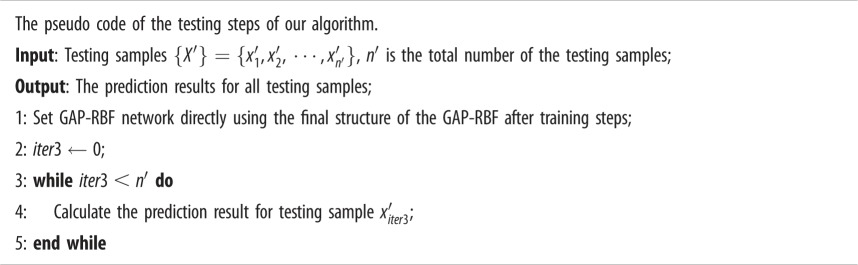


## 4. Experiment results and analysis

In this section, three classic forecasting issues about the age of abalone, the house price in Boston and the auto MPG are adopted for extensive test. The abalone problem is the estimation of the age of abalone from the physical measurements. The Boston house price problem is the prediction of median house price in the Boston area. The auto MPG problem is to predict the fuel consumption (miles per gallon) of cars in society. Note that abalone and auto MPG datasets are available in the repository of UCI machine learning and society predictions with non-uniform input distribution [29], and Boston house price dataset is available in the StatLib archive [30]. A brief description of these three datasets is given in table 1.

**Table 1.** The details of abalone, Boston house price and auto MPG datasets.

datasets	number of training samples	number of testing samples
abalone	3000	1177
Boston house price	481	25
auto MPG	320	78

**Figure 2.**
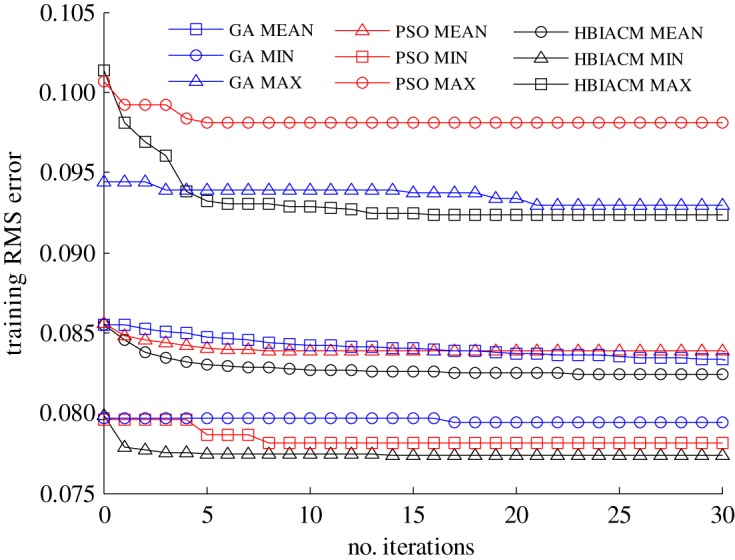
The comparison of training RMS error for three optimization algorithms (abalone dataset).

**Figure 3.**
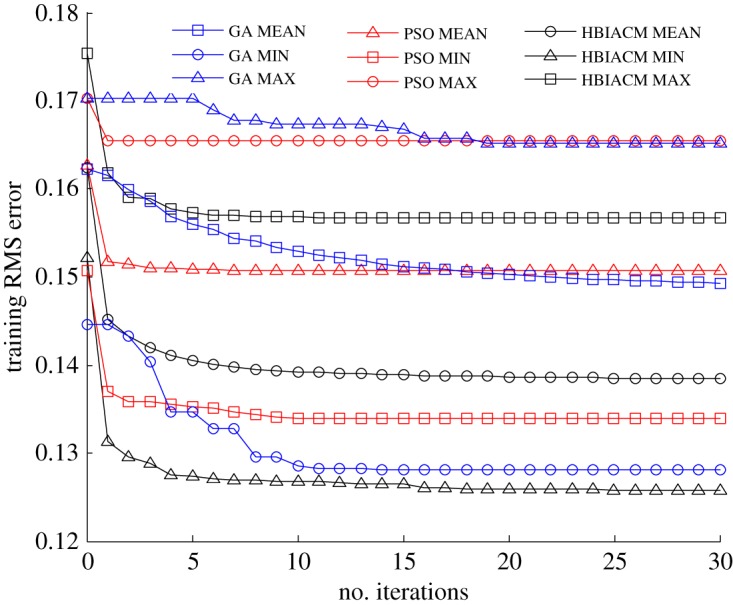
The comparison of training RMS error for three optimization algorithms (Boston house price dataset).

**Figure 4.**
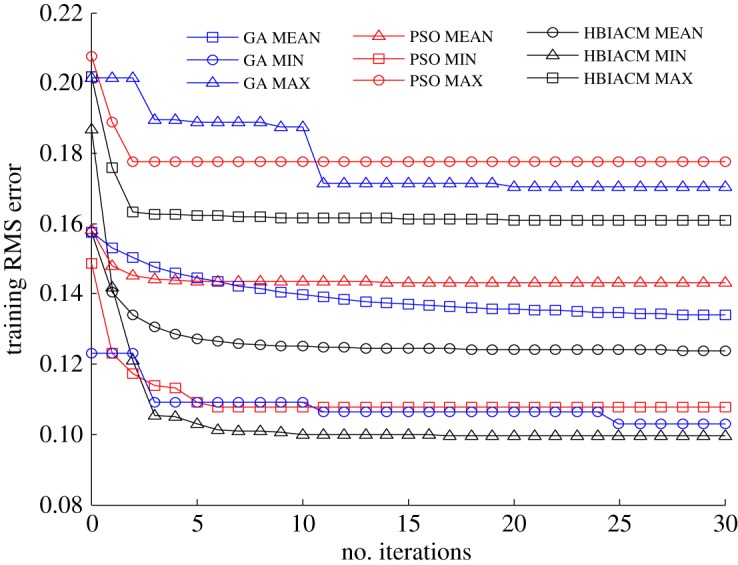
The comparison of training RMS error for three optimization algorithms (auto MPG dataset).

The abalone dataset includes 4177 samples, and each sample is composed of seven continuous input attributes, one nominal attribute and one integer output. The nominal attribute has three values including male, female, infant, and these three values are extremely non-uniformly distributed. For simplicity, the eight input attributes and one output can be normalized to the range [0, 1]^9^. Boston house price dataset includes 506 samples, and each sample comprises 12 continuous input attributes, one binary-valued attribute and one continuous output (median value of owner-occupied homes). The 13 input attributes and one output can be simply normalized to the range [0, 1]^14^. The auto MPG dataset includes 398 samples, and each sample is composed of seven inputs (four continuous ones and three multivalued discrete ones) and one continuous output (the fuel consumption). The seven input attributes and one output have been simply normalized to the range [0, 1]^8^. To demonstrate the effectiveness of HBIACM, we use it to optimize the weights and bias term between hidden layer and output layer of GAP-RBF network. In this experiment, we count the MAX, MIN and MEAN training root mean square (RMS) errors of 50 trails, and compare HBIACM with sole PSO or GA optimized GAP-RBF networks from figures 2 to 4 corresponding to abalone, Boston house price and auto MPG datasets, respectively. Specifically, the number of the training samples *n* is, respectively, set as 3000, 481 and 320 for these three datasets in the light of the given requirements for training. Subsequently, following the training steps of our algorithm (see the pseudo code of the training steps in §3.2), GAP-RBF is trained to obtain its final structure. Note that, in the iterative training process, the growing, pruning, and DEKF-based updating strategies are used first to get the intermediate structure including the number of hidden nodes, the weights and bias term between hidden layer and output layer, and then HBIACM is performed as an optimization method to obtain the optimized structure. In other words, we can achieve the final structure of GAP-RBF after fine tuning the network by HBIACM.

We can see from figures 2 to 4 that the training RMS error shows the similar varying trends for HBIACM, PSO and GA optimized GAP-RBF networks. That is, the training RMS error decreases with the increase of iterations. Note that, after 30 iterations, the MAX, MIN and MEAN training RMS errors of HIBACM are, respectively, lower than those of PSO and GA. That is to say, HBIACM achieves the better optimization results for GAP-RBF network, since it uses the competitive mechanism and combines the advantages of PSO and GA for network optimization.

Moreover, we compare the proposed method using or not using DEKF in GAP-RBF, MRAN, RANEKF and RAN networks. It has been known that so far there is no theoretical standard to choose the optimal values for the parameters of these networks, such as the expected error (for neuron growth and decrease), the pruning threshold, or the sliding window size for growing and pruning, etc. In this study, the experienced values for all of the parameters we used for comparison are extensively discussed by Huang *et al*. [14]. The commonly used parameters of GAP-RBF, MRAN, RANEKF and RAN are fixed as: the largest scale of interest in the input space *ɛ*_max_ = 1.15, the smallest scale of interest in the input space *ɛ*_min_ = 0.04, the overlap factor *κ* = 0.10, the decay constant *γ* = 0.999 and the desired approximation accuracy of the network output *e*_min_ = 0.0001. The rest of the parameters with respect to each algorithm are given in table 2. Note that, in this study, the basic parameters of our method are the same as those of GAP-RBF, and no other parameters need to be chosen.

**Table 2.** The parameters for each algorithm.

datasets	algorithms	parameters
abalone	RAN	*a* = 0.02
	RANEKF	*p*_0_ = 0.9, *q*_0_ = 0.00001
	MRAN	$e_{\min}^{\prime }=0.0001,\;M=50,\;p_{0}=0.9,\;q_{0}=0.00001$
	GAP-RBF	*S*(*X*) = 1, *p*_0_ = 0.9, *q*_0_ = 0.00001
Boston house price	RAN	*a* = 0.02
	RANEKF	*p*_0_ = 0.9, *q*_0_ = 0.00001
	MRAN	$e_{\min}^{\prime }=0.0001,\;M=80,\;p_{0}=0.9,\;q_{0}=0.00001$
	GAP-RBF	*S*(*X*) = 1.5657 × 10^−6^, *p*_0_ = 0.9, *q*_0_ = 0.00001
auto MPG	RAN	*a* = 0.02
	RANEKF	*p*_0_ = 0.9, *q*_0_ = 0.00001
	MRAN	$e_{\min}^{\prime }=0.0001,\;M=50,\;p_{0}=0.9,\;q_{0}=0.00001$
	GAP-RBF	*S*(*X*) = 0.0225, *p*_0_ = 0.9, *q*_0_ = 0.00001

In table 2, *a* is the learning factor, *p*_0_ is the initial value of error covariance matrix, *q*_0_ is the initial value of artificial process noise, $e_{\min}^{\prime }$ and *M* are the threshold and sliding window size for growing and pruning of MRAN, respectively, and *S*(*X*) is the size of the input space. Note that, for Abalone dataset, since the eight attributes of training samples are within the range [0, 1]^8^, the size of the input space *S*(*X*) is set as 1 for GAP-RBF. For Boston house price dataset, after directly analysing the distributions of the 13 input attributes, we find that the 13 input attributes are mainly non-uniformly distributed in the subspace [0, 0.1] × [0, 0.1] × ([0, 0.4]∪[0.6, 0.8]) × [0, 0.1] × ([0, 0.8]∪[0.9, 1.0]) × [0.3, 0.7] × [0.1, 1.0] × [0, 0.6] × ([0, 0.3]∪[0.9, 1.0]) × ([0, 0.5]∪[0.9, 1.0]) × ([0.2, 0.3]∪[0.4, 0.9]) × [0.9, 1.0] × [0, 0.8]. Therefore, the size of the input space can be simply estimated as *S*(*X*)=0.1 × 0.1 × 0.6 × 0.1 × 0.9 × 0.4 × 0.9 × 0.6 × 0.4 × 0.6 × 0.6 × 0.1 × 0.8 = 1.5676 × 10^−6^. For auto MPG dataset, *S*(*X*) can be simply estimated as 0.0225 using the same method. In our experiments, the performance comparison for each application has been done on 50 trials, and we summarize the results in terms of the mean values and the standard deviation (s.d.) values of training time (CPU time), training RMS error, testing RMS error, and the number of hidden units for each algorithm. All the simulations are carried out in Matlab 7.7 environment running in an Intel Core i3-3240, 3.4GHZ CPU and 4.0G memory PC. The mean and s.d. values of CPU time, training RMS errors, testing RMS errors and the number of hidden neurons with respect to the above three applications are denoted from tables 3 to 5, respectively. The training RMS error represents the fitting degree of the models to three given forecasting issues, and the testing RMS error reflects the forecasting accuracy. The CPU time and the number of the hidden neurons represent the real forecasting speed and final network structure of these six prediction algorithms, respectively.

**Table 3.** The performance comparison for abalone dataset.

algorithms	CPU time (s)		training RMS errors		testing RMS errors		no. hidden neurons	
	mean	s.d.	mean	s.d.	mean	s.d.	mean	s.d.
GAP-RBF+DEKF+HBIACM	*20.5078*	*3.6171*	*0.0820*	*0.0027*	*0.0828*	*0.0034*	*20.08*	*3.2315*
GAP-RBF+HBIACM	99.769	76.5411	0.0824	0.0021	0.0828	0.0032	19.72	3.3505
GAP-RBF	83.784	73.401	0.0963	0.0061	0.0966	0.0068	23.62	9.5081
MRAN	1500.4	134.08	0.0836	0.0039	0.0837	0.0042	87.57	7.1147
RANEKF	90 806	18 193	0.0738	0.0042	0.0794	0.0053	409.00	22.485
RAN	105.17	6.1714	0.0931	0.0091	0.0978	0.0092	345.58	12.578

From table 3 we can see that our method (GAP-RBF+DEKF+HBIACM) achieves better results with lower training RMS error and testing RMS error in comparison with GAP-RBF, MRAN and RAN. The training RMS error and testing RMS error of our method are just a little higher than those of RANEKF. The CPU time of our method is much faster than other five methods, and the number of the hidden neurons of our method is very small. In comparison with GAP-RBF, MRAN, RANEKF and RAN, our method obtains the smaller s.d. for CPU time, s.d. for training RMS error, s.d. for testing RMS error and s.d. for number of neurons, which means the stability of our method is better than the other four methods. In comparison with GAP-RBF+HBIACM, the speed of our method is enhanced through using DEKF, since DEKF decreases the size of error covariance matrix and saves the CPU time. From table 3, we can also see that the training RMS error, the testing RMS error and the number of neurons of GAP-RBF+HBIACM(s) with or without DEKF are similar through the different tests of random 50 trails.

From table 4, we can see that our method (GAP-RBF+DEKF+HBIACM) achieves the best testing RMS error in comparison with other five methods. Moreover, our method obtains the better training RMS error in comparison with GAP-RBF, MRAN, RAN, and the training RMS error of our method is just a little higher than that of RANEKF. For CPU time comparison, our method is faster than MRAN, RANEKF, RAN, and it is similar to GAP-RBF with the similar number of hidden neurons.

**Table 4.** The performance comparison for Boston house price dataset.

algorithms	CPU time (s)		training RMS errors		testing RMS errors		no. hidden neurons	
	mean	s.d.	mean	s.d.	mean	s.d.	mean	s.d.
GAP-RBF+DEKF+HBIACM	*1.7238*	*0.1126*	*0.1374*	*0.0064*	*0.1221*	*0.0382*	*5.96*	*0.8320*
GAP-RBF+HBIACM	2.5291	0.4252	0.1379	0.0126	0.1285	0.0350	4.54	0.6131
GAP-RBF	1.2399	0.2812	0.1507	0.0128	0.1418	0.0466	3.50	0.6468
MRAN	12.731	2.2585	0.1440	0.0108	0.1356	0.0411	13.58	1.8962
RANEKF	22.572	6.4159	0.1328	0.0086	0.1437	0.0464	19.98	1.8349
RAN	4.2664	0.4846	0.3449	0.0620	0.3432	0.0770	18.80	1.6413

From table 5, we can see that our method (GAP-RBF+DEKF+HBIACM) achieves the best testing RMS error, and the training RMS error of our method is just not as good as that of MRAN and RANEKF with 5 × 10^−4^ and 3 × 10^−4^ differences, respectively. For CPU time comparison, our method is faster than GAP-RBF+HBIACM and MRAN because of the utilization of DEKF, and it is slower than GAP-RBF, RANEKF and RAN. That is because in comparison with Abalone and Boston house price datasets, the size of training data in auto MPG dataset is relatively small, and the utilization of HBIACM in smaller dataset increases the CPU time.

**Table 5.** The performance comparison for auto MPG dataset.

algorithms	CPU time (s)		training RMS errors		testing RMS errors		no. hidden neurons	
	mean	s.d.	mean	s.d.	mean	s.d.	mean	s.d.
GAP-RBF+DEKF+HBIACM	*1.1266*	*0.0702*	*0.1091*	*0.0108*	*0.1126*	*0.0107*	*3.86*	*0.7001*
GAP-RBF+HBIACM	1.4308	0.1409	0.1097	0.0103	0.1177	0.0158	3.94	0.7398
GAP-RBF	0.4520	0.0786	0.1144	0.0132	0.1404	0.0270	3.12	0.7462
MRAN	1.4644	0.2453	0.1086	0.0100	0.1376	0.0226	4.46	0.7343
RANEKF	1.0103	0.1694	0.1088	0.0117	0.1387	0.0289	5.14	0.9037
RAN	0.8042	0.1417	0.2923	0.0808	0.3080	0.0915	4.44	0.8369

## 5. Conclusion

GAP-RBF network is a promising sequential learning algorithm for prediction analysis, since it has very few thresholds to define, and requires less memory. However, the selection of the parameters of such a network to deal with real-world applications is usually non-convex problem, which makes it difficult to handle. In this paper, a hybrid bioinspired intelligent algorithm is proposed to optimize the parameters of GAP-RBF. Specifically, the excellent local convergence of PSO and the extensive search ability of GA are both considered to optimize the weights and bias term, which are difficult to precisely optimize in basic GAP-RBF. Meanwhile, a competitive mechanism is introduced based on the respective advantage of PSO and GA to improve the search ability of the hybrid algorithm. That is, the population is partitioned in terms of the fitness of each individual, and the hybrid method can choose the appropriate individuals for effective search via the competitive mechanism. What is more, it has been known that the large number of inputs may cause the rapid growth of neurons and makes the error covariance matrix become huge. Such an undesirable process would finally result in an issue of computational overload. DEKF is introduced in this study by ignoring the cross-correlation terms between the nearest neuron and all other neurons in the error covariance matrix. Such a method reduces the size of error covariance matrix and decreases the computational complexity of the network. In our experiments, three classic forecasting issues, i.e. the predictions of abalone age, Boston house price and auto MPG, are adopted for extensive test, and the experimental results show that the proposed HBIACM performs better than single bioinspired optimization algorithms, e.g. PSO or GA, in the light of the MAX, MIN and MEAN training RMS errors. Moreover, through using DEKF, our method achieves the better results in comparison with the state-of-art sequential learning algorithms, including GAP-RBF, MRAN, RANEKF and RAN.

Currently, we are concentrating on the following extensions to the proposed method. First, we are now modifying our program in order to run it on GPU servers for performing high performance predictions. Second, based on the inherent implicit parallelism of PSO and GA, parallel computing techniques are adopted to optimize GAP-RBF network. For the third improvement, the extensive investigation for the variations of PSO and GA needs to be addressed in order to improve the optimization results and reduce training time. Finally, we are now expanding the capacity of our database by adding a large number of training datasets. Such expansions will support developing a GUI for users. It will be really nice if such a GUI can embed in our system, which will help users to perform various predictions. In addition, we are trying to develop an open interface to users for extensive test. The system will return a set of expected results on the basis of the feedback of users, which can be used to further evaluate and improve the performance of our system.

## Data accessibility

The abalone dataset is at http://archive.ics.uci.edu/ml/datasets/Abalone. The auto MPG dataset is at http://archive.ics.uci.edu/ml/datasets/Auto+MPG. The Boston house price dataset is at http://lib.stat.cmu.edu/datasets/boston.
